# Supplementary material for: Analysis of prevalence and influencing factors of stroke in elderly hypertensive patients: Based on the screening plan for the high-risk population of stroke in Jiading District, Shanghai
Source: PLoS One. 2021 Aug 9;16(8):e0255279. doi: 10.1371/journal.pone.0255279 (PMC8351920; doi:10.1371/journal.pone.0255279)
Supplement: Attachment — Submitted filename: rebuttal letter.docx [file pone.0255279.s003.docx]

**Dear Editor,**

We are pleased to hear that the manuscript is potentially acceptable for publication in your journal. Thank the editor and reviewers for valuable suggestions on our research. We also believe that this manuscript has been greatly improved after these revisions. We look forward to publishing our research in your journal. Point-by-point responses are listed below and have been numbered in this letter.

Yours truly,

The authors

***Journal Requirements:***

*Please review your reference list to ensure that it is complete and correct. If you have cited papers that have been retracted, please include the rationale for doing so in the manuscript text, or remove these references and replace them with relevant current references. Any changes to the reference list should be mentioned in the rebuttal letter that accompanies your revised manuscript. If you need to cite a retracted article, indicate the article’s retracted status in the References list and also include a citation and full reference for the retraction notice.*

Response:We have reviewed our reference list to ensure that it is complete and correct.We made the following changes to the references: the original references 2 and 9 were deleted and replace them with relevant current references.

***Reviewer #1:*** *Despite the revision, the authors did not manage to clarify how the study population was established in either the methods section in the manuscript or the response letter. As a consequence, I am not sufficiently convinced about the results interpretation and conclusion.
The statistics and results are relatively fine, but technical errors are universal (i.e. there is absolutely no way to investigate "risk" of an outcome in a cross-sectional design etc.) and must be corrected carefully before publication. Besides, results presentation is still unfortunately of low quality (inconsistent use of digit etc.)*

Response:We have previously explained in detail in our manuscripts and reply letters how our research population was obtained, and the details are as follows：Since 2016, a comprehensive screening of all chronic patients in the Chronic Disease Management Bank of Jiading District, Shanghai has been conducted, and the general survey was performed on a community basis. The plan is mainly based on the chronic disease registration management database of the district. It registers all diagnosed chronic disease patients in the district. According to the principle of "informed, consent, and voluntary", the health department and the community strengthen publicity and guidance and implement full coverage screening for people aged over 35, meeting the inclusion criteria, and with hypertension registered in the chronic disease management database. The inclusion criteria of survey objects include: 1) permanent residents whose annual residence time is ≥6 months, and are over 35 years old; 2) clearly diagnosed as hypertension patients; 3) no contraindications related to screening; 4) patients who can sign an informed consent form by themselves or accompanied by their guardians and participate in all screening projects voluntarily. As of 2019, 26174 hypertension patients were randomly selected as survey subjects. After excluding people who are unwilling to cooperate and do not meet the inclusion criteria, we finally screened 21,902 hypertension patients, with a screening response rate of 83.69%. For these screened populations, the potential influencing factors of stroke and the occurrence of stroke were recorded. In the end, a total of 18,724 people with hypertension 60 years and older were selected as our study population. At present, the screening project is still ongoing. According to statistics, there are 155,829 hypertensive patients in the chronic disease registration management database in the district. A lot of manpower, material, and financial resources are required to achieve a comprehensive screening. Therefore, valuable information could be obtained through the investigation and analysis of the first stage to guide the follow-up screening and crowd intervention.

In addition, I would like to thank you for your recognition of our statistics and results. In addition, as you said, our cross-sectional study has no way to get the risk of the outcome, so we use the effect index OR to describe our outcome.And we carefully checked the description of the results to avoid using RISK to describe the results. Of course, this is also one of the limitations of our research, so we explained this in the limitations section.

Finally, we carefully revised the results presentation, such as inconsistent use of digit.

***Reviewer #2:*** *My concerns had been fully addressed. No further revisions are necessary except language editing (minor revision)*

Response:Thank you for your recognition of our research. We have polished and carefully reviewed the language of the manuscript.
